# Cost-Effectiveness of the “Helping Babies Breathe” Program in a Missionary Hospital in Rural Tanzania

**DOI:** 10.1371/journal.pone.0102080

**Published:** 2014-07-09

**Authors:** Corinna Vossius, Editha Lotto, Sara Lyanga, Estomih Mduma, Georgina Msemo, Jeffrey Perlman, Hege L. Ersdal

**Affiliations:** 1 SAFER (Stavanger Acute Medicine Foundation for Education and Research), Stavanger University Hospital, Stavanger, Norway; 2 Research Institute, Haydom Lutheran Hospital, Haydom, Tanzania; 3 Ministry of Health and Social Welfare, Dar es Salaam, Tanzania; 4 Department of Pediatrics, Weill Cornell Medical College, New York, New York, United States of America; Seattle Childrens Hospital, United States of America

## Abstract

**Objective:**

The Helping Babies Breathe” (HBB) program is an evidence-based curriculum in basic neonatal care and resuscitation, utilizing simulation-based training to educate large numbers of birth attendants in low-resource countries. We analyzed its cost-effectiveness at a faith-based Haydom Lutheran Hospital (HLH) in rural Tanzania.

**Methods:**

Data about early neonatal mortality and fresh stillbirth rates were drawn from a linked observational study during one year before and one year after full implementation of the HBB program. Cost data were provided by the Tanzanian Ministry of Health and Social Welfare (MOHSW), the research department at HLH, and the manufacturer of the training material Lærdal Global Health.

**Findings:**

Costs per life saved were USD 233, while they were USD 4.21 per life year gained. Costs for maintaining the program were USD 80 per life saved and USD 1.44 per life year gained. Costs per disease adjusted life year (DALY) averted ranged from International Dollars (ID; a virtual valuta corrected for purchasing power world-wide) 12 to 23, according to how DALYs were calculated.

**Conclusion:**

The HBB program is a low-cost intervention. Implementation in a very rural faith-based hospital like HLH has been highly cost-effective. To facilitate further global implementation of HBB a cost-effectiveness analysis including government owned institutions, urban hospitals and district facilities is desirable for a more diverse analysis to explore cost-driving factors and predictors of enhanced cost-effectiveness.

## Introduction

Neonatal mortality is defined as death before one month of age and recent global estimates range from 2.9 to 3.6 million deaths per year [1]–[4]. Of these, as much as 50–70 percent may occur within the first day of life [1], [5]–[8]. Almost 99% of all neonatal deaths take place in resource-poor settings [1], [9]–[12]. A major factor contributing to the high mortality is a global lack of trained providers in neonatal stabilization and/or resuscitation. This is most acute in Sub-Saharan Africa with the highest neonatal mortality [13]. The context of “Helping Babies Breathe” (HBB) is based on the International Liaison Committee on Resuscitation (ICLOR) Consensus in Science recommendations. The program includes an evidence-based curriculum in basic neonatal care and resuscitation, utilizing simulation-based training to educate large numbers of birth attendants in low-resource countries [14]. The program was developed by the Global Implementation Task Force of the American Academy of Pediatrics. In September 2009, the Tanzanian Ministry of Health and Social Welfare (MOHSW) launched the National HBB program by implementing HBB training and data collection at eight study-sites in Tanzania. Haydom Lutheran Hospital (HLH) was the only rural site, located in the in the Manyara region in Northern Tanzania.

An evaluation of the HBB program with pooled data of all eight sites showed that early neonatal mortality (within the first 24 hours, ENM) was reduced significantly during the first year with a relative risk reduction of 42%, and that this reduction was sustained during the second year at 47%. Fresh stillborn (FSB) rates were significantly reduced in the second year, with a relative risk reduction of 24% [15].

The HBB program is especially developed for low resource settings. Recently, the United Nations promoted HBB as one of ten breakthrough innovations in order to close the Millennium Development Goal 4 gap before 2015 [16], and the HBB program will be implemented in many low-resourced countries around the world within the next years. However, a proper cost-effectiveness analysis (CEA) is missing. The implementation of the HBB program at HLH was closely linked to a descriptive observational open cohort study in the delivery room, initiated in August 2009 [17], [18]. In addition, it has been possible to obtain detailed cost information from the MOHSW and the research unit at HLH. Thus, we aimed at a CEA of the HBB program in a rural hospital in Tanzania by presenting the total costs to society per live saved and per life year gained and the separate cost factors.

## Methods

### The “Helping Babies Breathe” Training Program

HBB is an evidence-based curriculum in basic neonatal care and resuscitation, utilizing simulation-based training to educate large numbers of birth attendants in low-resource countries [14]. The course methodology focuses on hands-on practice using a simulator mannequin, emphazising the very first basic steps: drying, stimulation, suction, warmth, and initiation of bag mask ventilation within the “Golden Minute” after birth if indicated. The teaching tools are developed for efficient dissemination, and the educational kit contains a set of flip-over illustrations, an action plan, a neonatal simulator (NeoNatalie, Laerdal Medical), a student handbook, a manual resuscitator (Laerdal Medical), and a suction device (Pinguin, Laerdal Medical). The materials and equipment are left behind to facilitae re-training and dissemination.

*Haydom Lutheran Hospital* HLH is a faith-based organization (FBO), located in Northern Tanzania, at the Southern border of Mbulu district in the Manyara region, 300 km west of Arusha, which is the nearest urban center. The immediate catchment area includes about 500 000 people, while the greater reference area covers more than two million people. HLH is a missionary hospital. About 60% of the total funding is provided by the Norwegian government. In addition to a Norwegian managing medical director there is also a varying number of staff from Western countries working there in long-term or short-term appointments. The hospital is a 420-bed hospital owned by the Mbulu Diocese of the Evangelical Lutheran Church in Tanzania, and is fully incorporated in the national health plan under the MOHSW. The hospital provides surgical, medical, gynecological, comprehensive emergency obstetric, and basic emergency newborn care. Since 2009, HLH has offered free transport service for delivering women with an increase in annual number of deliveries from 3000 in 2008 to 5000 in 2011.

### Implementation of the HBB Training Program at HLH

National implementation of HBB in Tanzania started in September 2009 and has been facilitated by a Health Ministry Commitment and integration into the Health Care System. The program was launched with two days HBB trainings of 40 Master Instructors selected from University and Referral hospitals [15] to start a cascade model where Master Instructors could train Trainers of Trainees (ToTs) who again would train health care providers. In April 2010 the initial one-day HBB course was held at HLH. Only half of the birth attendants were able to attend and no local Master Instructors were trained to facilitate continuation of HBB re-trainings. An evaluation of data from HLH revealed no improvements in mortality during the first months after the initial HBB course [19]. An additional study, testing skills and knowledge among birth attendants at HLH showed that both knowledge and technical skills were improved when rated in a simulated setting seven months after the initial one-day HBB course as compared to before, but this improvement did not transfer into clinical practice. The number of babies being suctioned and/or ventilated at birth did not change, and the use of immediate stimulation decreased after the initial HBB training [20]. Therefore, in February 2011, a Master Instructor, representative from the MOHSW, returned to HLH and trained six ToTs who started in-situ low-dose-high-frequency (LDHF) HBB trainings in the labor ward: The newborn simulator (NeoNatalie, Lærdal, Norway) was placed in the labor ward easily accessible for frequent practicing in addition to weekly training sessions conducted by ToTs of about 30 minutes during working hours. Finally, a second full-day HBB course took place in May 2011 where most of the staff from Maternity participated. Consequently to the establishment of LDHF-training the number of infants being stimulated increased and the need for bag mask ventilation decreased with a corresponding decline in ENM [19].

### Data collection – ENM and FSB

In August 2009, closely linked to the national HBB program, an ongoing descriptive observational open cohort study was initiated in the delivery room at HLH [17], [18]. Fourteen research assistants/observers were trained to observe the birth attendants' performances related to delivery, newborn management, and perinatal outcome. The findings are recorded on a data collection form following every delivery. The observers work in three shifts over 24 hours. Three observers cover each shift; two are always located in the labour ward or in the theatre during caesarean sections; one in the adjacent neonatal area.

The HBB program was fully implemented at HLH in February 2011, when the LDHF training was initiated by local ToT. To calculate the effectiveness of the training program, we compared ENM and FSB during the 12 months before the complete implementation (01.02.2010–31.01.2011) and the 12 months after (01.02.2011–31.01.2012). We hypothesized that without the training program the rate of ENM and FSB would be the same during both years. No other interventions were implemented and the number of deliveries and staff were stable. The number of lives saved was thus calculated by the estimated number of deaths during the 12 months after the HBB implementation given the same mortality as during the 12 months before implementation minus the observed number of deaths during this period.

### Data collection – costs

Costs for the initial one-day HBB course in April 2010 were borne by the Tanzanian MOHSW, and cost information was provided by the Principle Investigator for the National HBB program (GM) in the MOHSW. Costs for the second one-day HBB course in May 2011 and the LDHF HBB re-trainings were borne by HLH, and the number of participants, number of trainers and cost details were provided by the research department at HLH. Costs for training material were given by Lærdal Global Health.

The initial training of 40 Master Instructors in 2009 did not include personnel from HLH, therefore Master Instructors form this pool had to travel to HLH to conduct the first HBB course. These Master Instructors have delivered HBB courses in their own hospitals but also around the country. The cost of training these 40 initial Master Instructors were not included.

All costs are expressed in USD with an exchange rate of 1 USD = 1350 TZS for April 2010 and of 1 USD = 1510 TZS for May 2011.

For the evaluation of the cost-effectiveness in an international context, total costs were converted to International Dollars (ID) based on the purchasing power parity (PPP) as given by the World Health Organization (WHO) for 2005 [21].

### Statistics

The software program PASW 20.0 (SPSS Inc; Chicago, USA) was used for statistical analysis. Independent-samples t-test was used to compare means of continuous parametric variables. The relation between categorical variables was explored by Chi-square tests. Two-sided p-values lower than 0.05 were considered statistically significant. The number of live years gained was based on the number of lives saved and the life expectancy at birth in Tanzania of 55.4 years as given by UN-DESA [22]. Disease adjusted life years (DALYs) were calculated based on the calculations outlined by Fox-Rushby and Hansons [23]. As recommended there, calculations are based on the life expectancy in the observed population (55.44 years). We calculated DALYs based on two assumptions: i) Discounting rate (r) = 0.03 per year (implying that years that might be gained in the future have less value than years gained right now) and age weighting with K = 1 and β = 0.04 (assigning a lower number of DALYs to years lived at young and older ages); ii) no discounting, no age weighting (equivalent to life years gained).

### Ethics statement

The ongoing observational study at the delivery room at Haydom Lutheran hospital that forms the basis for the measurement of effectiveness in the present study, received the following approval by The Regional Committee for Medical and Health Research Ethics, Western Norway: The committee considers the project (reference number 2009/302) to be “an evaluation program among certified health care workers with standardized anonymous collection of related routine data on patient outcomes.” Formal approval from a Norwegian ethical committee is thus not required. Informed consent was not obtained. The National Institute for Research in Tanzania approved of the observational study as part of the research project “Towards: MDG 4&5: Implementing “Helping Babies Breathe” and “Helping Mothers Survive” to improve perinatal and maternal outcome at Haydom Lutheran Hospital (reference number NIMR/HQ/R.6a/Vol IX/1247).

As the presented cost-effectiveness analysis does not involve any confidential or personal data, an ethics statement from the Regional Committee for Medical and Health Research Ethics, Western Norway was not required and thus not applied for. The National Institute for Research in Tanzania was informed about the extension of the research project and approved the study (reference number NIMR/HQ/R.6c/Vol II/172) and as well of the publication of the results (reference number NIMR/HQ/R.12/Vol XIV/15).

All authors participated in the collection of the cost data: Costs borne by the Tanzanian Ministry of Health and Welfare were provided by Georgina Msemo and Jeffrey Perlman. Costs borne by Haydom Lutheran Hospital were provided by Editha Lotto, Sara Lyanga, Estomih Mduma and Hege L.Ersdal. Costs for the training material were collected by Corinna Vossius, who also coordinated the collection of cost data.

## Results

### Lives saved and live years gained

As shown in table 1 there were 4876 deliveries during the 12-month observation period before full HBB implementation, and 4734 deliveries during the 12-month period after full implementation, for a total of 9610 deliveries. The ENM decreased significantly from 11.1 to 7.2 deaths per 1000 deliveries (p = 0.047) in the period after full HBB implementation, while the decrease in FSB was not statistically significant.

**Table 1:** Comparison of the 12-months observation period before and after full HBB implementation.

Time Period	01.02.10–29.01.11	01.02.11–31.01.12	P-value*
Deliveries	N = 4876	N = 4734	
ENM/1000	11.1/1000 (n = 54)	7.2/1000 (n = 34)	0.047
FSB/1000	16.0/1000 (n = 78)	14.4/1000 (n = 68)	0.517
Stimulation	N = 704 (14.4%)	N = 758 (16.0%)	0.032
BMV	N = 352 (7.2%)	N = 259 (5.7%)	0.003

*Pearson Chi-Square analysis, 2-sided.

ENM = Early neonatal mortality; BMV = Bag mask ventilation.

Based on the ENM of 11.1/1000 the expected number of deaths during the post-implementation period was 53 deaths, while the observed number was 34. As the difference in FSB was not significant, we assumed that no lives were saved due to averted FSB. Hence, the total number of lives saved was 19.

Based on a live expectancy at birth of 55.4 years the total number of potential life years gained was 1052.6 years.

### Costs

Costs for the initial one-day HBB training in April 2010 were USD 2084, while it was USD 1515 for the one-day HBB re-training in May 2011. The training of four local ToT in February 2011 was linked to research activity. Therefore, only costs for one day accommodation amounted to USD 20. Table 2 presents details about the different costs factors. Costs for training material amounted to USD 812. Once implemented, the LDHF re-training incurred no direct costs as it was done during working hours and with the existing training material.

**Table 2:** Total direct costs and cost factors of HBB training at HLH.

	Total costs and cost factors in USD		Payer
**Initial HBB training April 2010**	**Total costs**	**2084**	**MHSW**
	Travel expenses^a^	1248	
	Trainers' allowance	89	
	Participants' allowance	474	
	Administration^b^	273	
	Substitutes; 64 participants, one working day^c^	0	
**Training of four local Trainers of Trainees February 2011**	**Total costs**	**20**	**HLH**
	Travel expenses^a^	20	
	Trainers' allowance	0	
	Participants' allowance	0	
	Administration^b^	0	
	Substitutes; four participants, one working day^c^	0	
**Refresher HBB course May 2011**	**Total costs**	**1515**	**HLH**
	Travel expenses^a^	790	
	Trainers' allowance	139	
	Participants' allowance	331	
	Administration^b^	255	
	Substitutes, 53 participants, one working day^c^	0	
**LDHF training**	**Total costs**	**0**	**HLH**
	Trainers' allowance	0	
	Working time 30 minutes once per week^c^	0	
**Material**	**Total costs**	**812**	**Donation**
	8 NeoNatalies à USD 70	560	
	8 Resuscitators à USD 15	120	
	8 Penguin suctions à USD 3	24	
	4 Flip charts à USD 27	108	
	Maintenance	0	
**Overall costs (%)**	**Total costs**	**4431 (100)**	
	Travel expenses^a^	*2048 (46)*	
	Trainers' allowance	*228 (5)*	
	Participants' allowance	*805 (18)*	
	Administration^b^	*528 (12*	
	Material	812 (18)	

a Travel expenses include transport,accommodation and per diems.

b Administration includes as well refreshments during the course.

c Courses took place during normal working hours and no extra staff was hired during the courses.

MHSW = Tanzanian Ministry of Health and Social Welfare; HLH = Haydom Lutheran Hospital; LDHF = Low-dose-high-frequency.

Hence, the total costs for the full HBB implementation was USD 4431.

### Costs per life saved, per life year gained and per DALY averted

Costs per live saved were USD 233, while it was USD 4.21 per life year gained (table 3).

**Table 3:** Costs per life saved, life year gained and DALY averted.

Unit	n	Costs per unit
**Implementation and maintenance costs included**		
Lives saved	16	USD 233
Life years gained	1053	USD 4.21
**Only maintenance costs included**		
Lives save	|19	USD 80
Life years gained	1053	USD 1.44
**DALYs averted [0.03,1,0.04]**	578	ID 22.75
**DALYs averted [0,0,0]**	1053	ID 12.49

DALYs [r, K, β] = Disability adjusted life years [discounting rate, age weighting constant K, age weighting constant β].

We regard costs for the initial HBB course and the training of local Master Instructors as pure implementation costs, while the LDHF training and one HBB refresher course per year are regarded crucial for maintaining knowledge, skills, and commitment to the HBB program. As the LDHF incurs no direct costs, expenses arise only for the one-day HBB refresher course of USD1515 per year. Thus, once the program is implemented costs are USD80 per life saved and USD 1.44 per life year gained.

The total number of DALYs averted was 578 DALYs if age weighting and discounting was applied and 1053 without age weighting and discounting. Converted to ID, the costs would amount to ID 22.75 per DALY averted and ID 12.49 per DALY averted, respectively for the two scenarios (table 3). To range the costs of an intervention per DALY, the WHO discerns three categories of cost-effectiveness: Highly cost-effective (less than gross domestic product (GDP) per capita); Cost-effective (between one and three times GDP per capita); and Not cost-effective (more than three times GDP per capita) [WHO-CHOICE; 23]. As the GDP per capita in Tanzania is ID 2154 the HBB program can be ranged as highly cost-effective.

### Sensitivity analysis (Table 4)

**Table 4:** Sensitivity analysis.

	Costs	Costs per life saved	Costs per life year gained
Presented costs*	**4431**	**231**	**4,21**
Staff salary included	+2384	345	6,22
Travel costs excluded	−2048	125	2,26

	Lives saved		
**Presented risk reduction for ENM of 35.1%**	19	**231**	4,21
Risk reduction of ENM 20%	10	443	8,06
Risk reduction of ENM 10%	5	886	16,11

*All costs in USD.

At HLH, no substitutes were hired to replace the staff participating in the full-day HBB courses. However, we assume that this might be necessary at other sites. Alternatively, participants may attend the course in their spare time and have to be paid extra. Therefore, the sensitivity analysis includes wages for one working day per participant (64 participants in 2010 and 53 in 2011) in addition to the LDHF training of 30 minutes per week, increasing costs by 53%.

On the other hand, as HLH is a very rural and remote hospital. Travel expenses amounted to almost half of the total costs as trainers had to come from larger hospitals like Bugando in Mwanza, about 400 kilometers away. Avoiding travel expenses in a more urban setting and without the need to provide competent instructors from out of state would thus reduce costs by 46%.

We assumed a number of 19 lives saved, based on a risk reduction of ENM of 35.1%. In the sensitivity analysis we present as well a cost-effectiveness analysis with a risk reduction of 20% and 10%, respectively. However, even assumed a risk reduction of only 10%, costs per DALY averted (including discounting and age weighting) would amount to ID 87 and thus still be in the range of highly cost-effective measures.

## Discussion

A retrospective evaluation of the costs-effectiveness of the HBB program in a rural FBO hospital in Northern Tanzania revealed costs of USD 233 per life saved and USD 4.21 per life year gained. However, costs for maintaining the program were significantly lower and are estimated to be USD 80 per life saved and USD 1.44 per life year gained.

With costs of about 12 to 23 ID per DALY averted, the HBB program can be considered highly cost-effective. These costs per DALY are comparable to a number of other measurements for maternal and neonatal health listed by WHO-CHOICE for Sub-Saharan countries such as the community newborn care package (8 ID per DALY averted) or support for breast feeding mothers (ID 10 per DALY) [24]. It is also comparable to the Essential Newborn Care Course that focuses on the first 7 days after birth. A CEA of this course in urban Zambia states costs per live saved of USD 208 and USD5.24 per life year gained, while costs for maintaining the program were USD1.84 per life year gained [25]. However, comparisons across various CEAs might be impaired by different methodologies regarding items included or not included into the cost analysis.

The implementation of the HBB program at HLH started with a one-day HBB course. Though this led to an improved performance in simulation of skills and knowledge [20], a decrease in ENM was first seen only after full implementation with systemized LDHF [19]. We therefore assume that there is a certain threshold for training input – both resource and money-wise - before the implementation of new health measurements pays off, while any training below this threshold might not yield much benefit. However, maintenance costs for the training program are significantly lower than the initial implementation.

We describe the costs as they presented in realtime at HLH. Importantly, in contrary to the WHO CHOICE guidelines [26], these costs did not comprise wages, as the courses were attended during working hours and no substitutes were hired. However, the need for substitutes might raise the costs by about 50 percent, and even more in an urban setting where wages are higher. On the other hand, HLH is a rural hospital, and the Master Instructors had to travel far distances and be accommodated. Thus, travel expenses were the biggest cost driver. In an urban setting where competence is more readily available, costs are likely to be significantly lower.

The major strengths of this study are its linkage to the observational study in the delivery room, securing reliable outcome data, and the meticulous registration of training activities and costs by the research department of the hospital. On the other hand, the single center design is a limitation of the study. About five thousand births per year might not yield enough statistical power to prove changes due to the program. The rural setting at a missionary FBO hospital might not be transferrable to other sites, and a CEA at a public urban hospital might yield quite different results. In addition, we are not aware of any other interventions during the observation period, however, there might be confounding factors contributing to a decrease in ENM that are not captured in this study. ENM and FSB during the pre-implementation phase were lower than the national average [15], possibly resulting in a lower reduction in ENM and FSB and thus lower effectiveness. Furthermore, we had no data about morbidity. The calculation of DALYs is solely based on mortality data, not taking into account that an increased resuscitation activity might lead to an increased number of children with hypoxic brain damage. However, data from both the observational study and the national study reveal a significant decrease in the need of bag mask ventilation, indicating that the increase in immediate stimulation prevented development of severe asphyxia. In addition, at HLH a 40% reduction in 24 hour mortality was noted, without a corresponding increase in deaths beyond 24 hours and with a 50% reduction in admissions to neonatal area after full HBB implementation [27]. We therefore assume that morbidity due to hypoxic brain damage was not increased.

## Conclusions

The number of children five years and younger dying world-wide has been reduced from 12 million per year in 1990 to seven to eight million in 2011. Vaccination programs have played an important role in this progress. However, to reduce mortality further early newborn mortality has to be addressed. The HHB program has been proven highly effective with a reduction of ENM of nearly fifty percent at eight sites in Tanzania. This study of a rural missionary FBO hospital reveals that the program is as well highly cost-effective measures in a low-resource setting. However, to facilitate further global implementation of the program an analysis of the cost-effectiveness in a multi-center setting including urban and government owned hospitals, is necessary, exploring cost-driving factors and predictors for enhanced cost-effectiveness.
